# The Duplicitous Origin of Ovarian Cancer

**DOI:** 10.5041/RMMJ.10106

**Published:** 2013-01-30

**Authors:** Gil Mor, Ayesha Alvero

**Affiliations:** Department of Obstetrics Gynecology and Reproductive Sciences, Reproductive Immunology Unit, Yale University School of Medicine, New Haven, CT, USA

**Keywords:** Ovarian cancer, chemotherapy, cancer markers, cancer stem cells

## Abstract

The past few decades have seen many advances in the treatment of a variety of cancers. Unfortunately, for ovarian cancer, which is the most lethal type of gynecologic malignancy, no new therapeutic approach has been successfully introduced since the 1990s. Ovarian cancer is usually detected in later stages, when remission rates are high and tumors are resistant to chemotherapy. Little is known about the primary lesion in ovarian cancer. Recently, it has been shown that the origin of ovarian cancer can be cells from adjacent tissue or cells from other primary tumors, which make their way to the ovaries due to the unique nature of their microenvironment during ovulation. The tumor in ovarian cancer is heterogeneous and hierarchically organized. In this review, we discuss the role of ovarian cancer stem cells in the process of tumor formation and recurrence. We propose the need to shift the paradigm away from the classification of ovarian cancer as a single disease with a single cellular origin. Understanding the complexity of the disease will facilitate devising new methods for fighting this cancer and improving the life of many women inflicted with the disease.

## INTRODUCTION

Approximately 22,000 new cases of ovarian cancer are diagnosed each year in the United States, and around 16,000 women die each year from ovarian cancer.^1^ A woman’s lifetime chance of being diagnosed with ovarian cancer is 1/72 with a 1% chance of dying from this disease. Platinum-based chemotherapy drugs against ovarian cancer were introduced almost 30 years ago, and the last big breakthrough was the introduction of the taxane drugs in the early 1990s. These drugs increased the mean survival rate from 24 to 38 months,^2^ that is, they did not cure the disease but rather increased the patient’s life-span by several months. Currently, the common course of treatment for ovarian cancer is to perform a complete debulking in which the woman’s internal reproductive organs are removed. Debulking is followed by chemotherapy with drugs such as carboplatin and taxol. Roughly 80% of the women respond to chemotherapy, but the relapse rate is very high with a 60% relapse rate within 1 year and 80% within 5 years.^3^ When a relapse occurs, the disease is usually resistant to chemotherapy and spreads throughout the abdomen. When the disease reaches this stage, there is no weapon in medicine’s arsenal that can save such a patient. For this reason, epithelial ovarian cancer is considered the most lethal type of gynecological cancers today.^4^

As in many cancers, early detection is critical for survival. If ovarian cancer is detected early, during stages I and II, the survival rate is quite good. However, the majority of ovarian cancers are detected at stages III and IV, where the 10-year survival rate is between 10% and 20%.^5^ Early detection of ovarian cancer is difficult, partly due to its non-specific symptoms and lack of a screening test for the general population. As a result, diagnosis often occurs when the disease is at an advanced stage and when the prognosis is poor. A considerable amount of research has gone into finding early detection markers for ovarian cancer, but all have failed in clinical trials since they did not detect all the ovarian cancer cases. Some markers were able to detect 80% of the cases, but if there is a substantial number of false negatives and the markers do not detect 100% of the cancer cases, the markers are considered failures.^6^,^7^ These failures, in early detection and therapy, prompted the question whether we really understand the etiology and the biology of this disease.

## THE ORIGIN OF OVARIAN CANCER

**Figure 1:**
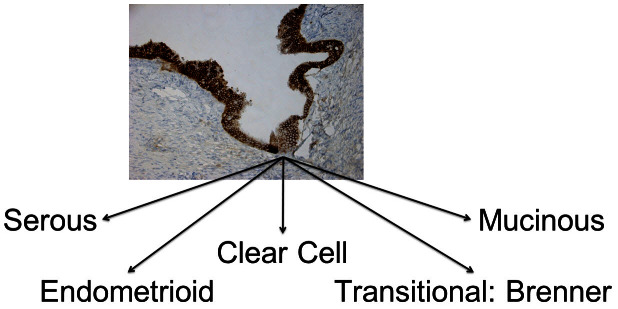
**Histologically different types of ovarian cancer.**

One fundamental question that has yet to be answered is the origin of ovarian cancer. In spite of numerous studies, the original lesion that gives rise to ovarian cancer has thus far not been identified. Some researchers even considered the original lesion to be created *de novo*.^8^ The prevalent theory is that ovarian cancer originates from the surface epithelium layer of the ovaries, which is of mesothelial origin. The epithelial cells involute inside the ovaries and form cysts. Subsequently, due to an accumulation of genetic mutations, the cells turn cancerous and a tumor is formed. The problem with this theory is that there are different types of ovarian cancers. These subtypes include endometrial ovarian cancers, clear cell carcinomas, and mucinous, serous, and Brenner transitional tumors, whose cellular make-up is not necessarily mesothelial in nature (Figure 1).^9^ All these cancers have diverse histological origins and different clinical and pathological behaviors. Therefore, it is unlikely that all these tumors originate from the same cell or the same lesion. The simplistic theory of the origin of ovarian cancer is even more improbable if we take into account that most of the disparate cancer cell types are not ovarian in origin.^10^

While it was logical to assume that the genesis of the ovarian tumor is the ovary, it is also logical that the progenitor cells of the ovarian tumors originate from tissues adjacent to the ovary, such as the fallopian tubes.^11^ Studies in which fallopian tubes were more carefully examined confirmed that small *in-situ* early invasive tubal carcinomas occur in women with a genetic predisposition for ovarian cancer.^12^ In addition, 70% of sporadic (non-hereditary) ovarian and peritoneal high-grade serous carcinomas demonstrated mucosal tubal involvement, including serous tubal intraepithelial carcinoma (STIC).^13^ Further support for this argument is the finding that nearly all STICs overexpress p53, similar to high-grade serous carcinoma. Laser capture micro-dissection studies have demonstrated that these lesions harbor mutated p53. In addition, STICs that are associated with a concomitant ovarian carcinoma shared not only morphologic features but also identical p53 mutations, indicating a clonal relationship.^13^ Therefore, it seems very likely that there is a “two-way traffic” between the ovaries and the fallopian tubes. Normally, the oocytes travel from the ovaries to the fallopian tubes, but in certain cases cells from the fallopian tube may travel to the ovaries and contribute to malignancies.

These observations have opened the possibility to revise the theory on the origin of ovarian cancer; it is plausible that the initial lesion might not be of ovarian origin but a metastasis of abnormal or transformed cells from different sites in the peritoneum. What these cells are and how they reach the ovaries are two main questions that need to be addressed and which will be examined in this review.

## HETEROGENEITY OF CANCER CLONES

**Figure 2:**
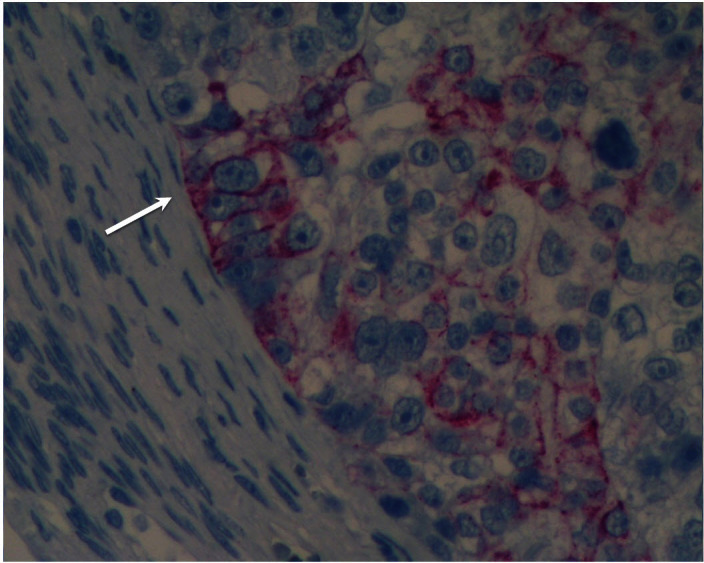
**Two distinct cell types in an ovarian tumor.** The large cells are positive for CD44 staining (arrow), and the smaller flanking cells are negative for CD44 staining.

The classical or clonal model to explain tumor formation and progression suggests that a tumor originates from a single mutated cell that is no longer under normal cell cycle control and thus divides incessantly. The constantly dividing cell forms a mass of identical fast-dividing cells, which results in a tumor. However, while this classical clonal paradigm indeed exists, it provides a partial description of the biology of the tumor. Increasing evidence suggests that the tumor is more complex; indeed it has been shown that there is a hierarchy in the cancer cells, where progenitor cancer stem cells, also known as tumor-initiating cells, can produce two or more distinct cell types. When we evaluated the cellular composition of epithelial ovarian tumors we were able to identify at least two distinct subtypes of ovarian cancer cells: the classical cancer cells, characterized by small size and fast cell division which we refer to as type II ovarian cancer cells; and the type I ovarian cancer cells, with different morphological characteristics and distinctive behavior (Figure 2). The type I cells are slowly dividing cells and share many markers with pluripotent stem cells. These markers include CD44, MyD88, ALDH1, and others, which the smaller, rapidly dividing subtype lacks. Type I cells can rebuild the original tumor in mice (*tumor-initiation potential*), give origin to CD44-negative/MyD88-negative type II cells (*differentiation capacity*), serve as tumor vascular progenitors (*pluripotency*), and are chemoresistant.^14^–^18^ Indeed, levels of type I cells are associated with shorter progression-free survival in ovarian cancer patients.^18^ Our findings are in line with other studies that have shown the existence of tumor-initiating cells in ovarian cancer through the use of different markers suggestive of the heterogeneity of the disease.^14^,^19^–^23^

An important characteristic of type I cells is their potential to give origin to the fast-dividing type II cells. This aspect was demonstrated in our animal studies where only CD44-positive cells are able to form tumors in nude mice. Once the tumor was established, we evaluated the cellular composition of the tumors. If the clonal hypothesis had been correct, the tumor would have been comprised of only CD44-positive cells. However, the results showed that only about 10% of the cells were CD44-positive, while the majority of the cells were molecularly and morphologically different (CD44-negative type II cells).^14^,^24^ Tumor formation does not occur if we inject type II cells in nude mice, indicating that the capacity to establish a heterogeneous tumor is a unique property of type I cells.^14^

**Figure 3:**
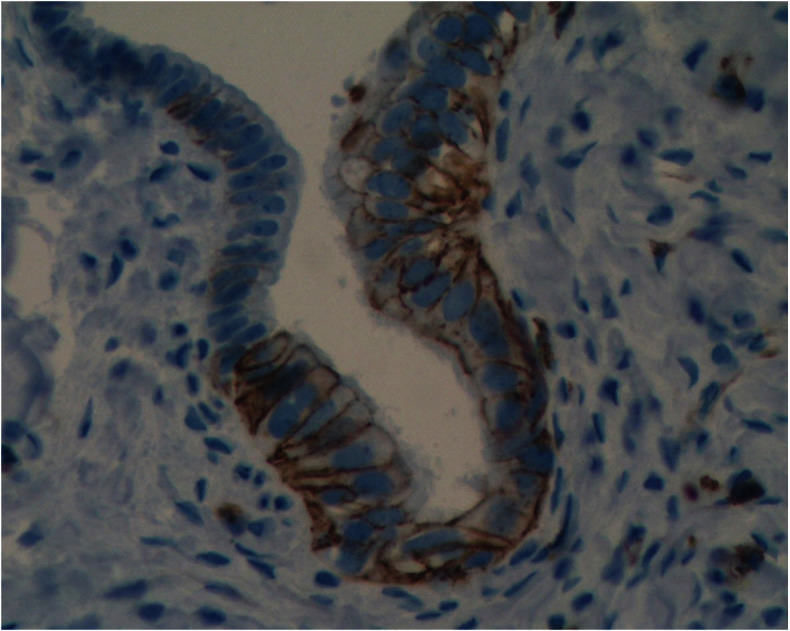
**High expression of CD44 seen in cells of the fallopian tube.** Note the morphologic differences between the CD44 positive and CD44-negative cells.

**Figure 4:**
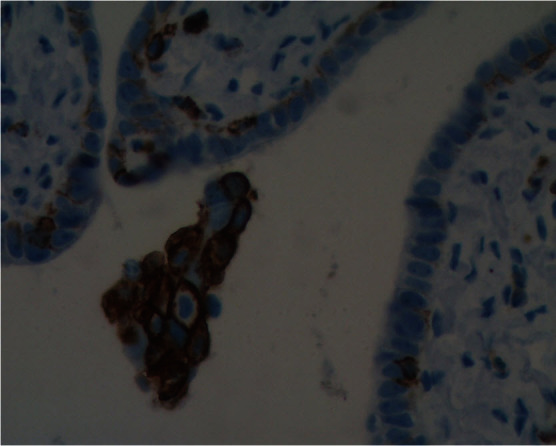
**CD44-positive cells from the fallopian tube that broke away from the tissue.**

Pluripotent progenitor cancer cells can be the source of the heterogeneous tumors seen in ovarian cancer. One possible source for these cells, based on stem cell markers and p53 signature, is the epithelium of the fallopian tubes. However, for the fallopian tube to be the source of ovarian cancer, cells from its epithelial layer must first detach, survive without attachment to the basement membrane, and acquire mobility to travel to the ovaries. We found CD44-positive cells in the fallopian tubes with morphological characteristics different from the rest of the epithelium (Figure 3). In addition, we observed CD44-positive cells “shed” by the epithelium, with morphological characteristics of migratory cancer stem cells (Figure 4). These observations suggest that migratory cancer stem cells might originate from the fallopian tubes or from other sites of the female reproductive tract and travel through the fallopian tubes before reaching the ovaries.

## THE OVARIES AS TARGETS FOR CANCER CELLS

**Figure 5:**
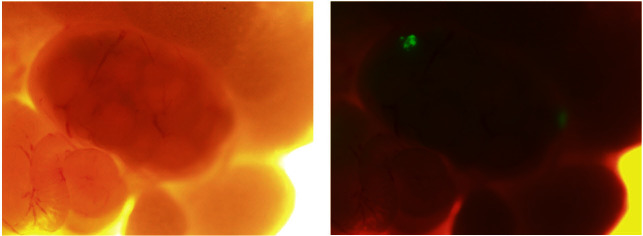
**Attachment of CD44-positive ovarian cancer cells to the mouse ovary.** The left side is a light microscopy picture of an ovary. The right side is a fluorescent microscope picture of the same ovary showing the cancer progenitor cells (green) attached to the ovary.

One of the main factors associated with the prevention of ovarian cancer is the use of hormonal contraception. A potential physiologic explanation for this association is ovulation and inflammation. Many studies have linked inflammatory processes and cancer.^16^ High levels of cytokines and chemokines induced by inflammation can induce tumorigenesis and metastasis.^25^ Inflammatory processes during ovulation represent an important reason that the ovaries are susceptible to developing tumors. During ovulation, the mature follicle ruptures the surface of the ovary due to inflammatory processes.^26^ This inflammatory condition can be detected by the high levels of cytokines and chemokines secreted in the follicular fluid and produced by the ovary. The ovulation sites are micro-wound sites on the ovary’s surface, and through these sites the cancer cells can enter the ovaries and form an ovarian tumor (Figure 5). Observations from our laboratory suggest that migratory cancer progenitor cells may be attracted to the ovaries by the inflammatory process; furthermore, the ovulatory environment creates a fertile soil for these cells to attach and proliferate, leading to the formation of a “local” ovarian tumor. These observations support the hypothesis of an extra-ovarian origin of some forms of ovarian cancer. Thus, this may explain the failure to find a single specific marker for the early detection of ovarian cancer. Presently, studies have focused on finding biomarkers associated with the epithelial cells of the ovaries or serum markers associated with the ovarian microenvironment. If indeed ovarian cancer can originate from various types of transformed cells from multiple sites in the female reproductive tract, then each type of ovarian cancer would have its own set of distinctive markers. Identification of these extra-ovarian progenitors of ovarian cancer is a first step in the recognition of possible markers for early detection.

## A MULTI-TARGET APPROACH TO TREATING OVARIAN CANCER

As previously mentioned, chemotherapy is standard for women with ovarian cancer. In most cases, the cancer responds to the chemotherapy, cancer markers substantially decrease, and a state of remission is achieved. However, for the majority of the women with ovarian cancer, a short period of remission is followed by a relapse, in which the tumor is chemo-resistant to both taxol and carboplatin. To address the question of recurrence and chemo-resistance we evaluated the phenotype of cells that survive chemotherapy and found that these cells possess the markers that distinguish the type I cells or tumor-initiating cancer cells. CD44-positive cells did not respond to chemotherapy and instead grew steadily even in the presence of these drugs. These results further support the current consensus that CD44-positive cells represent the chemo-resistant phenotype.^19^,^21^,^22^,^27^–^29^ On-going studies using an *in-vivo* recurrence model of ovarian cancer also showed that tumors initially respond to chemotherapy and shrink considerably. However, after a short remission period the tumor grows back, and at this stage it is totally non-responsive to chemotherapy treatment that was previously successful. Moreover, the cellular composition of the recurring tumor differs significantly from the primary tumor. These studies clearly indicate that the two distinct subtypes of ovarian cancer cells must be treated differently. If only one type of treatment is given, recurrence and disease progression will be delayed but not prevented. Subsequent tumors will be comprised of a different cell type that will only respond to an entirely different agent.

## CONCLUSION

**Figure 6:**
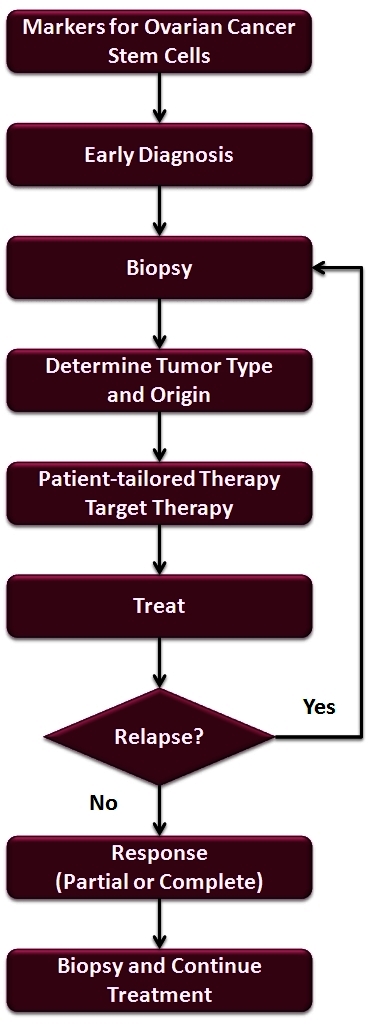
**Flow chart for diagnosing and treating patients with ovarian cancer.**

There are multiple forms of epithelial ovarian cancer due to the diversity of the cells that form the primary lesion. Therefore, a wider range of early detection markers should be used to screen for early detection of the disease. In addition, treatment for ovarian cancer should take into account the clonal diversity that is persistently found in the ovarian tumor. The future treatment for patients with ovarian cancer will include multiple steps: Once a tumor is detected, a biopsy should be taken and the cell type and origin should be determined. A personalized tailor-made chemotherapeutic regimen should be provided based on the tumor’s unique cellular make-up (Figure 6). Maintenance should be done by targeting the surviving cancer stem cells, consequently preventing relapse. If relapse occurs, novel treatment options are needed to treat recurrent disease, which differ from conventional chemotherapy since numerous studies have shown that these cells are resistant to chemotherapy. Once again, the selection of the therapy should be carried out based on the determination of the cellular composition of the recurrent tumor; this would then increase the possible effectiveness of the selected agents. Only then can we expect to see a marked improvement in the response and survival rate of ovarian cancer.

## Abbreviations:

STIC: serous tubal intraepithelial carcinoma

## References

[1] Jemal A, Siegel R, Ward E, Hao Y, Xu J, Thun MJ (2009). Cancer statistics, 2009. CA Cancer J Clin.

[2] Kumar S, Mahdi H, Bryant C, Shah JP, Garg G, Munkarah A (2010). Clinical trials and progress with paclitaxel in ovarian cancer. Int J Womens Health.

[3] SEER Cancer Statistics Review, 1975–2005.

[4] Seo JM, Park S, Kim JH (2012). Leukotriene B4 receptor-2 promotes invasiveness and metastasis of ovarian cancer cells through signal transducer and activator of transcription 3 (STAT3)-dependent up-regulation of matrix metalloproteinase 2. J Biol Chem.

[5] Kosary KL, Ries LAG, Young JL, Keel GE, Eisner MP, Lin YD, Horner M-J (2007). Chapter 16: Cancer of the Ovary. SEER Survival Monograph: Cancer Survival Among Adults: U.S. SEER Program, 1988–2001, Patient and Tumor Characteristics. SEER Program, NIH Pub. No. 07-6215.

[6] Visintin I, Feng Z, Longton G (2008). Diagnostic markers for early detection of ovarian cancer. Clin Cancer Res.

[7] Kim K, Visintin I, Alvero AB, Mor G (2009). Development and validation of a protein-based signature for the detection of ovarian cancer. Clin Lab Med.

[8] Bell DA, Scully RE (1994). Early de novo ovarian carcinoma. A study of fourteen cases. Cancer.

[9] Lee KR, Young RH (2003). The distinction between primary and metastatic mucinous carcinomas of the ovary: gross and histologic findings in 50 cases. Am J Surg Pathol.

[10] Kelemen LE, Köbel M (2011). Mucinous carcinomas of the ovary and colorectum: different organ, same dilemma. Lancet Oncol.

[11] Kurman RJ, McConnell TG (2010). Precursors of endometrial and ovarian carcinoma. Virchows Arch.

[12] Shih IeM, Chen L, Want CC (2010). Distinct DNA methylation profiles in ovarian serous neoplasms and their implications in ovarian carcinogenesis. Am J Obstet Gynecol.

[13] Kurman RJ, Shih IeM (2010). The origin and pathogenesis of epithelial ovarian cancer: a proposed unifying theory. Am J Surg Pathol.

[14] Alvero AB, Chen R, Fu HH (2009). Molecular phenotyping of human ovarian cancer stem cells unravels the mechanisms for repair and chemoresistance. Cell Cycle.

[15] Alvero AB, Fu HH, Holmberg J (2009). Stem-like ovarian cancer cells can serve as tumor vascular progenitors. Stem Cells.

[16] Leizer AL, Alvero AB, Fu HH (2011). Regulation of inflammation by the NF-kappaB pathway in ovarian cancer stem cells. Am J Reprod Immunol.

[17] Mor G, Yin G, Chefetz I, Yang Y, Alvero A (2011). Ovarian cancer stem cells and inflammation. Cancer Biol Ther.

[18] Steffensen KD, Alvero AB, Yang Y (2011). Prevalence of epithelial ovarian cancer stem cells correlates with recurrence in early-stage ovarian cancer. J Oncol.

[19] Bapat SA, Mali AM, Koppikar CB, Kurrey NK (2005). Stem and progenitor-like cells contribute to the aggressive behavior of human epithelial ovarian cancer. Cancer Res.

[20] Szotek PP, Pieretti-Vanmarcke R, Masiakos PT (2006). Ovarian cancer side population defines cells with stem cell-like characteristics and Mullerian inhibiting substance responsiveness. Proc Natl Acad Sci U S A.

[21] Moserle L, Indraccolo S, Ghisi M (2008). The side population of ovarian cancer cells is a primary target of IFN-alpha antitumor effects. Cancer Res.

[22] Curley MD, Therrien VA, Cummings CL (2009). CD133 expression defines a tumor initiating cell population in primary human ovarian cancer. Stem Cells.

[23] Gao Q, Geng L, Kvalheim G, Gaudernack G, Suo Z (2009). Identification of cancer stem-like side population cells in ovarian cancer cell line OVCAR-3. Ultrastruct Pathol.

[24] Alvero AB, Montagna MK, Craveiro V, Liu L, Mor G (2012). Distinct subpopulations of epithelial ovarian cancer cells can differentially induce macrophages and T regulatory cells toward a pro-tumor phenotype. Am J Reprod Immunol.

[25] Raman D, Sobolik-Delmaire T, Richmond A (2011). Chemokines in health and disease. Exp Cell Res.

[26] Espey LL (1980). Ovulation as an inflammatory reaction--a hypothesis. Biol Reprod.

[27] Kurrey NK, K A, Bapat SA (2005). Snail and Slug are major determinants of ovarian cancer invasiveness at the transcription level. Gynecol Oncol.

[28] Zhang S, Balch C, Chan MW (2008). Identification and characterization of ovarian cancer-initiating cells from primary human tumors. Cancer Res.

[29] Curley MD, Garrett LA, Schorge JO, Foster R, Rueda BR (2011). Evidence for cancer stem cells contributing to the pathogenesis of ovarian cancer. Front Biosci.

